# "Concordance between comorbidity data from patient self-report interviews and medical record documentation"

**DOI:** 10.1186/1472-6963-8-85

**Published:** 2008-04-16

**Authors:** William Corser, Alla Sikorskii, Ade Olomu, Manfred Stommel, Camille Proden, Margaret Holmes-Rovner

**Affiliations:** 1 College of Nursing, Michigan State University, B500A West Fee Hall., East Lansing, Michigan 48824, USA; 2 College of Human Medicine, Michigan State University, B338 Clinical Center, East Lansing, Michigan 48824, USA; 3 Emergent BioSoultions, 3500 N. Martin Luther King Blvd., Lansing, Michigan 48906, USA; 4 Center for Ethics and Humanities in the Life Sciences, Michigan State University, C208 East Fee Hall, East Lansing, Michigan 48824, USA

## Abstract

**Background:**

Comorbidity is an important adjustment measure in research focusing on outcomes such as health status and mortality. One recurrent methodological issue concerns the concordance of comorbidity data obtained from different reporting sources. The purpose of these prospectively planned analyses was to examine the concordance of comorbidity data obtained from patient self-report survey interviews and hospital medical record documentation.

**Methods:**

Comorbidity data were obtained using survey interviews and medical record entries from 525 hospitalized Acute Coronary Syndrome patients. Frequencies and descriptive statistics of individual and composite comorbidity data from both sources were completed. Individual item agreement was evaluated with simple and weighted kappas, Spearman Rho coefficients for composite scores.

**Results:**

On average, patients reported more comorbidities during their patient survey interviews (mean = 1.78, SD = 1.99) than providers had documented in medical records (mean = 1.27, SD = 1.43). Higher proportions of positive responses were obtained from self-reports compared to medical records for all conditions except congestive heart failure and renal disease. Older age and higher depressive symptom levels were significantly associated with poorer levels of data concordance.

**Conclusion:**

These results demonstrate that survey comorbidity data from ACS patients may not be entirely concordat with medical record documentation. In the absence of a gold standard, it is possible that hospital records did not include all pre-admission comorbidities and these patient survey interview methods may need to be refined. Self-report methods to facilitate some patients' complete recall of comorbid conditions may need to be refined by health services researchers.

**Trial Registration:**

ClinicalTrials.gov NCT00416026.

## Background

Many researchers have demonstrated the influence of different combinations of comorbid conditions on patient outcomes such as quality of life, depression, and death [1-4]. While the phenomenon of composite comorbidity is considered a complex function of the number and severity of pre-existing health conditions a hospital patient possesses, there is currently no *gold standard *to measure their composite *comorbidity *[5,6].

Although health services researchers have debated the specific causal pathways of different comorbidity combinations on health outcomes, researchers have less frequently tested the concordance of comorbidity data obtained from various reporting sources, [5-8] calling for further investigation [2,5,7-11]. Providers and researchers need to identify the more rigorous collection methods concerning patient-level comorbidity data for the formulation of patients' treatment plans and research concerning their subsequent health outcomes [2,5,8,9].

In the literature, researchers have identified three primary sources for obtaining patient-level comorbidity data: a) administrative diagnosis databases or clinical patient registries,[12,13] b) medical records,[9,10] and c) patient self-reports [11,14-17]. Due to the frequently missing data in many administrative diagnosis databases, experts have suggested that patient medical records may be a more complete source of comorbidity data [2,5,14,16].

Still, medical records possess limitations related to: a) inconsistent or absent documentation standards, b) limited availability of recent documentation, and c) underreporting of pre-admission conditions judged by providers to be less pertinent to patients' admitting diagnoses [10,11,15]. Medical record notes also often contain elements of both patient self-report and earlier provider documentation, sometimes offering a hybrid source of original data [10,11,18].

### Medical Record Source

One of the most frequently advocated methods used to generate comorbidity data from medical records is the Charlson Comorbidity Index (CCI),[19,20] which has been tested with large samples in numerous settings [1,5,6,8]. A patient's composite CCI score is calculated as a weighted sum of the presence of 19 documented health conditions such as *Congestive Heart Failure, Diabetes*, or *Peripheral Vascular Disease*. The CCI method was originally developed and tested with a sample of 607 medical patients, with weights assigned to different conditions derived from relative risk estimates of 30-day breast cancer mortality obtained from proportional hazards models [19]. Researchers have obtained similar predictive results concerning patient complications and functional outcomes for cardiac, diabetes, and depression samples [1,4,6,8].

### Patient Self-Report Source

As an alternative to medical records, patient may be asked about their comorbidities in mailed surveys or individual telephone or face-to-face interviews [9,11,14,21]. The primary difference from medical records is that survey methods result in "unfiltered" self-reports, (e.g. self-reports that are not filtered through the additional questioning of healthcare personnel). Researchers have demonstrated patient self-reports to be a generally reliable data source through careful rewordings of more complex individual comorbidity items [9-11,14,17,21].

In 1996, Katz, et al. [20] tested a self-report comorbidity source method comprised of a brief questionnaire which includes the same overall series of comorbidity items as the CCI [19,20]. Initially, hospital patients were asked if they had experienced each health condition before their current admission, with some questions reworded using more recognizable diagnosis terms (e.g. *Chronic Pulmonary Disease *converted into *Asthma*, *Emphysema*, and *Chronic Bronchitis*, or *Acute Myocardial Infarction *(AMI) reworded to *Heart Attack*). Several additional questions are asked whether patients take medications for certain conditions to prompt patients' recall [21]. The individual item weightings for the Katz [21] method are almost entirely the same as for the CCI [19,20].

### Comparison of CCI and Katz Comorbidity Data Patterns

In the original Katz et al study, test-retest reliability coefficients were assessed by intra-class correlation, with estimates of 0.91 obtained from the self-report questionnaire compared to 0.92 for the CCI method [21]. Spearman correlation coefficients between composite scores from the two sources were somewhat weak at 0.63, and even smaller for less-educated patients. In 1996, Katz et al estimated the cost of obtaining each patient's comorbidity data to be $0.93 dollars per mailed questionnaire, $1.67 per self-report interview, versus $3.50 per medical record audit [21].

Individual condition data obtained concerning diagnoses such as *Diabetes, Hypertension, Stroke*, and *Cancer *from various self-reporting *forms *have demonstrated consistently higher levels of concordance [9,10,22]. Other conditions such as *Heart Failure, Arthritic Conditions*, or *Pulmonary Conditions *have beenless concordant in elderly or veteran patient samples [9,10,22]. Some studies have failed to show that socio-demographic characteristics such as increased age, marital status, or completed education levels exert significant influences on concordance levels between the reporting methods [10,15]. Other studies have found that younger age, female gender, and/or more completed education significantly increased concordance levels [9,22].

#### Purpose Statement

The purpose of the prospectively planned study analyses was to examine the concordance levels between comorbidity data generated from Acute Coronary Syndrome (ACS) patients using two related self-report and medical record sources. Rather than treating either source as the gold standard, the authors examined the relative concordance between these two methods and patient predictors affecting concordance levels. The following research questions were addressed:

1. "What is the level of concordant validity of individual item responses and composite comorbidity scores obtained from ACS patient self-report interviews and medical record documentation sources?"

2. "Which patient characteristics predict discordance between comorbidity data obtained from ACS patient self-report interviews and medical record documentation?"

Before the analyses, the authors had hypothesized that the self-report source would provide more complete data concerning certain comorbid conditions affecting patients' daily post-discharge health and function Due to the specific ACS admitting diagnosis under which patients were admitted, the authors also anticipated that medical record notes would reflect a general underreporting of conditions assessed by hospital providers to be less pertinent to patients' cardiac diagnoses. Based on the findings of earlier groups, [10,22] they further expected that the examination of data concordance from ACS patients would provide a more homogenous sampling framework than using data from patients admitted with multiple diagnoses or comorbid conditions.

## Methods

### Sample

The primary randomized intervention study from which these data were obtained enrolled a total sample of 719 ACS patients who had been hospitalized in five mid-Michigan hospitals [23]. Study inclusion criteria included a working admission diagnosis of ACS, age ≥21 years, possession of a functional home telephone, and documented serum Troponin I levels ≥ established upper normal limits during index hospitalization. Exclusion criteria were: inability to speak English and/or complete phone interviews, or discharge to a non-home setting. Nurse recruiters recruited patients between January 14, 2002 and April 13, 2003. The results of this primary study have already been published [4,24-27].

Data regarding patients' comorbidities were collected after discharge from both: a) structured baseline telephone patient interviews (N = 525) using the Katz [21] self-report method, and b) hospital medical record audits (N = 710) using the original CCI [19,20] method with paper or electronic medical records (approximately 50% in each form). Baseline interviews were conducted after index hospitalization (Mean = 14.11 days, SD = 9.6), by trained interviewers at a Michigan State University survey institute using a structured telephone interview protocol. Before any patient data were collected, approval had been obtained from the institutional review boards on campus and each participating hospital. Data collectors were blinded to patients' study group status.

All medical record data were collected by a group of study nurses who had been oriented to the chart auditing manual and protocols by the study Community Project Manager. Comorbidity data were obtained from medical record: 1. face sheets, 2. history and physical reports, and 3. admission and discharge notes. Chart auditors entered data onto a standard data collection form and referred to the chart audit manual concerning specific comorbidity data fields. Additional string field data were entered for several rare health conditions not included in the CCI [19,20] framework. Periodic chart auditor meetings were conducted to review chart audit protocols, and the results of redundant chart audits were reviewed by the community project manager to confirm the overall data entry reliability of newer auditors. The Community Project Manager continued to sample each reviewer's charts to maintain quality control. Reliability ≥98% was maintained throughout. Errors were largely omitting items in the history and physical, with little bearing on the study data set. This high level of inter-rater reliability is higher that many studies using the CCI [19,20] method. Interclass correlation coefficients of standardized chart audits typically range from 0.83 to 0.93, likely due to consistent supervision in our study [28,29].

Patient socio-demographic data collected by interview included: patient age category, gender, race (categorized into *White *or *Non-White*), and education completed (categorized into *less than high school*, *high school *graduate, *or at least some college or more*). Clinical measures included: a) level of pre-admission physical activity from the *Activity Status Index*,[30] and b) level of pre-admission depressive symptoms from the *Center for Epidemiologic Studies-Depression *(CESD) [31].

### Data Set Characteristics

Of the total of 719 enrolled patients, 525 (73.0% of enrolled) patients completed a post-discharge baseline interview to offer almost complete self-report socio-demographic and comorbidity data. Patients who consented, but did not participate in the baseline interview were more likely to have received anti-anxiety medications (OR: 2.58, p < 0.01), and were more likely to be minorities using US census categories (OR: 2.02, p < 0.01). Fifteen post-discharge deaths were identified from State of Michigan vital records; mortality was not a major contributor to attrition.

Less than 1% of the baseline interviewed patients refused to answer any comorbidity item, or indicated that they were unsure whether they possessed a condition. Only comorbidity data documented in one of the primary medical record locations were included in these analyses. No distinction was made between missing or refused data obtained from either source. Concordance analyses were limited to the 525 sample patients who provided comorbidity data from both reporting sources.

### Data Concerns

Due to our *ACS working admission diagnosis *inclusion criterion, all patients were documented as have recently sustained an AMI. Still, due to patients' recent cardiac admission possibly biasing their recall of having sustained an AMI, composite comorbidity scores were evaluated from each source with and without this item included.

### Analyses

Descriptive summary statistics were used to describe the comorbidity data from both reporting sources. Composite comorbidity scores from both sources were calculated, and compared using matched t-tests. In addition, Spearman Rho coefficients between composite comorbidity scores were estimated.

To analyze concordance between individual comorbidity items, we calculated the proportion of total agreement (on both reported presence and absence), and kappa coefficients with 95% confidence intervals for each condition. McNemar's tests [32,33] were used to test the concordance between the two data sources. Finally, a series of multivariate logistic regression models were run using age, gender, completed education, and depressive symptoms as predictors of agreement or disagreement in reporting patterns from the two methods for individual comorbidity items [34]. Due to the inadequate cell frequencies, the race variable was not included in the models.

The logistic regression models evaluated the probability of comorbidities appearing in either one source but not the other versus the agreement of both sources. The logistic regression analysis was conducted with adjustment for clustering of patients within hospitals using a generalized estimating equations approach with a compound symmetry correlation matrix [35,36]. All statistical tests were two-sided and performed using S.A.S. version 9.1 [37].

## Results

Study recruiters completed a mean number of 2.44 (SD 1.87) contacts with 1,707 patients who had been initially assessed by recruiters to be eligible, with 988 of these patients either refusing to participate (419 or 24.5%) or assessed to be too sick to enroll (569 or 33.3%). Table 1 lists the major socio-demographic and clinical characteristics of the 525 patients who provided comorbidity data from both reporting sources.

**Table 1:** Demographic & Clinical Characteristics of Patients at Baseline (n = 525)

**Variable**	**N**	
**Age at admission**	**525**	**M = 59.73 **(SD 12.00)
**Gender**	**525**	
Male		**334 **(63.6%)
Female		**191 **(36.4%)
**White/Non-White Race**	**525**	
White		**443 **(84.4 %)
Non-White/Multiracial/Other		**82 **(15.6 %)
**Current Marital Status**	**525**	
Married		**350 **(66.7%)
Divorced/Separated/Widowed		**174 **(33.1%)
**Work for Pay of Profit?**	**524**	
Yes		**226 **(43.0%)
No		**298 **(56.8%)
**Completed Education**	**521**	
Less than High School		**99 **(18.9%)
High school diploma		**196 **(37.3%)
Some college or more		**230 **(43.8%)
**Family Income**	**467**	
Less than $15,000 per year		**113 **(25.0%)
$15,000 or more per year		**354 **(75.8%)
**Activity Status Index **[26] (scale 0 – 54.55)	**525**	**M = 29.56 **(SD 17.21)
**CESD Depression **[27] (scale 0 – 60)	**524**	**M = 13.56 **(SD 10.48)
**Ejection Fraction**	**452**	**M = 50.19 **(SD 12.93)

Only 132 (25%) patients had a documented ejection fraction of less than 45%. Notably, although 154 (29.3% of total sample) of patients reported having sustained a heart attack during their baseline interviews, only 126 (81.8% of these same patients) had a *prior AMI *documented in their medical records.

Table 2 lists the descriptive statistics of the composite scores obtained from the two reporting sources, their quartiles, and Spearman coefficients. The magnitude of Spearman correlation coefficient with and without AMI included reflected disagreement of the two sources that was confirmed by the comparison of the mean composite scores. The means were significantly higher for the self-report method (p < .01), both with and without the AMI item included. The effect sizes for the difference in means between the two methods were .31 with AMI included, and .29 with AMI excluded. According to Cohen's classification,[38] these effect sizes are considered medium. Therefore, highly statistically significant disagreement was not simply due to large sample size, but reflected a practically important difference.

**Table 2:** Composite Comorbidity Score Data Patterns

**Variable**	**Mean (St Dev)**	**Median**	**Q1**	**Q3**	**Spearman rho**	**T (P)**
Charlson [18,19] composite score (prior AMI included) *	1.27 (1.43)	1	0	2	0.57 (p < .01)	7.12 (<.01)
Katz [20] composite score (prior AMI included) *	1.78 (1.99)	1	0	3		
Charlson [18,19] composite score (prior AMI excluded)	1.03 (1.30)	1	0	2	0.50 (p < .01)	6.73 (<.01)
Katz [20] composite score (prior AMI excluded)	1.49 (1.82)	1	0	2		

* (possible range of 0 (i.e. no comorbid conditions) to 37 (maximum possible number of comorbid conditions)

**Figure 1:**
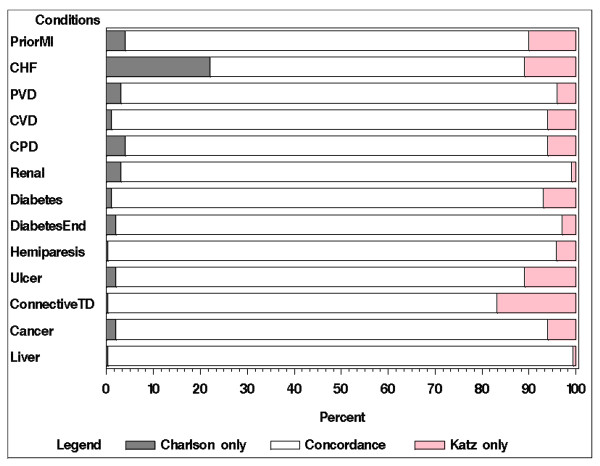
**Rates of agreement and disagreement of individual comorbidity items from two data sources.** NOTE: the relative proportions of comorbid conditions vary.

Table 3 displays the patterns of individual comorbidity item data derived from the two sources, as well as Kappa coefficients, percents of overall agreement, and p-values of McNemar's test [31,32] levels of agreement. Only two comorbid conditions were more frequently reported in patient medical records: *Congestive Heart Failure/Heart Failure *(p < .01) and *Renal/Kidney Disease (p = .13)*. All other conditions were more frequently reported by patients than documented in the medical records, with the differences in reporting rates reaching statistical significance for all conditions except *Peripheral Vascular Disease *and *Diabetes with End-organ Damage*. Figure 1 displays rates of agreement and disagreement between the two sources. With exception of AMI, the kappa agreement coefficients reflected fair to poor agreement between comorbidity data obtained from the two sources [38].

**Table 3:** Individual Comorbidity Item Agreement

**Comorbid Condition**	**Charlson Medical Record N Yes (%)**	**Katz Self-report N Yes (%)**	**Kappa (95% CI)**	Overall Percent Agreement*	**P-Value of McNemar's test**
Prior AMI/Heart attack	126 (24)	154 (29.33)	0.63 (0.56, 0.70)	86	<0.01
Congestive heart failure	150 (28.6)	95 (18.1)	0.09 (0.01, 0.18)	67	< .01
Peripheral vascular disease	28 (5.3)	37 (7.1)	0.43 (0.27, 0.58)	93	0.13
Cerebrovascular disease	31 (5.9)	56 (10.7)	0.54 (0.41, 0.67)	93	< 0.01
Chronic pulmonary Disease/Asthma bronchitis	45 (8.6)	59 (11.2)	0.43 (0.30, 0.55)	90	0.06
Renal/Kidney disease	24 (4.6)	17 (3.2)	0.47 (0.28, 0.66)	96	0.13
Diabetes	116 (22.1)	142 (27.1)	0.80 (0.72, 0.86)	92	< 0.01
Diabetes with end-organ damage	23 (4.4)	26 (5.0)	0.42 (0.24, 0.60)	95	0.56
Hemiparesis	3 (0.6)	24 (4.6)	0.14 (-0.04, 0.32)	96	<0.01
Ulcer disease	30 (5.7)	74 (14.1)	0.29 (0.17, 0.41)	87	< 0.01
Connective Tissue Disorder/Arthritis-Rheum. Arthritis	5 (1.0)	92 (17.5)	0.07 (0.00, 0.13)	83	< 0.01
Dementia	0 (0)	4 (0.8)			
Any type of cancer	26 (5.0)	44 (8.4)	0.33 (0.18, 0.48)	92	<.01
Liver Disease (mild to moderate)	1 (0.2)	3 (0.6)		99	

* overall percent agreement was defined as the number of concordant counts (both answered "yes" or both answered "no" in two sources) divided by the total sample size and expressed as a percent.

Tables 4 lists the odds ratios of disagreement between the two reporting sources versus their agreement for individual comorbidity items with prevalence of at least 3% in each data source. The reported odds ratios are adjusted for age, depressive symptoms, and level of completed education. Since gender had an insignificant association with the concordance of any individual comorbidity items, it was not included in the final model. For many comorbid conditions, increased age was significantly associated with an increased probability of disagreement between the two sources.

**Table 4:** Odds Ratios (ORs) and 95% confidence intervals for ORs of comorbid conditions documented in medical record but not in patient self report or in self-report but not documented (relative to agreement of both sources): effects of patient age, education and depressive symptoms.

**Comorbid Condition**	**Presence in**	**Age**	**Education **>= High school versus < High school	**CES-D**
Prior AMI	medical record but not in self report	1.03 (0.99, 1.06)	1.37*** (0.44, 4.32)	1.03 (1.00, 1.07)
	self report but not in medical record	1.03 (1.01, 1.06)**	0.46 (0.24, 0.89)*	1.01 (0.98, 1.04)
Congestive heart failure	medical record but not in self report	1.01 (0.99, 1.02)	0.62 (0.36, 1.06)	1.00 (0.98, 1.02)
	self report but not in medical record	1.03 (1.01, 1.05)*	0.44 (0.23, 0.84)*	1.03 (1.00, 1.05)
Peripheral vascular disease	medical record but not in self report	1.02 (1.98, 1.07)	0.42 (0.13, 1.41)	1.01 (0.96, 1.07)
	self report but not in medical record	1.01 (0.98, 1.05)	1.02 (0.32, 3.20)	0.99 (0.95, 1.04)
Cerebrovascular disease	medical record but not in self report	1.04 (0.97, 1.11)	0.57 (0.09, 3.48)	1.01 (0.93, 1.09)
	self report but not in medical record	1.04 (1.01, 1.07)*	1.14 (0.46, 2.84)	1.04 (1.01, 1.08)*
Chronic pulmonary disease	medical record but not in self report	1.01 (0.98, 1.05)	0.47 (0.17, 1.27)	1.01 (0.97, 1.06)
	self report but not in medical record	1.01 (0.98, 1.04)	1.31 (0.51, 3.38)	1.04 (1.00, 1.07)*
Renal disease	medical record but not in self report	1.10 (1.05, 1.15)**	2.56 (0.50, 12.97)	1.01 (0.95, 1.07)
	self report but not in medical record	1.03 (0.97, 1.09)	0.22 (0.04, 1.08)	1.02 (0.96, 1.10)
Diabetes	medical record but not in self report	1.08 (1.02, 1.16)*	0.65 (0.13, 3.20)	1.08 (1.01, 1.15)*
	self report but not in medical record	1.04 (1.01, 1.07)**	1.47 (0.57, 3.83)	1.03 (1.00, 1.07)
Diabetes with end-organ damage	medical record but not in self report	1.08 (1.03, 1.14)**	0.84 (0.23, 3.09)	1.06 (1.01, 1.12)*
	self report but not in medical record	1.05 (1.00, 1.09)*	0.47 (0.16, 1.45)	1.04 (0.99, 1.09)
Ulcer disease	medical record but not in self report	1.01 (0.96, 1.06)	0.68 (0.17, 2.72)	1.00 (0,95, 1.06)
	self report but not in medical record	1.02 (1.00, 1.05)	0.79 (0.40, 1.56)	1.02 (0.99, 1.04)
Any type of cancer	medical record but not in self report	1.01 (0.96, 1.05)	0.57 (0.16, 2.02)	1.01 (0.96, 1.06)
	self report but not in medical record	1.06 (1.03, 1.09)**	3.72 (1.05, 3.12)*	1.04 (1.01, 1.08)*

** p < .01

*p < .05

*** For example, OR = 1.37 for the level education reflects the fact that the odds of prior AMI documented in medical record but not in self report relative to being documented in both sources were 37% higher for those with at least high school education compared to those with less than high school education.

The odds of medical record documentation of diabetes and renal disease when the patient him/herself does not report these conditions increased with age (with respective odds ratios of 2.59 and 2.16 relative to agreement for each additional 10 years of age). At the same time, the odds of a patient reporting diabetes, a prior heart attack, congestive heart failure, cerebrovascular disease and cancer not documented in the medical record also increases with age. Only in the case of diabetes do we see both types of disagreement increasing with age.

The effect of depressive symptoms was somewhat similar to age with higher symptom scores associated with a higher probability of disagreement. This association reached statistical significance for increased patient self-reports of cerebrovascular disease, chronic pulmonary disease, and cancer. The effect of completed education reached statistical significance for two conditions, but did not show a consistent direction of effect (the magnitude of the odds ratios was very different across multiple items).

## Discussion

These results indicate that the concordance of ACS patient comorbidity data from patient survey interviews and medical record documentation sources may certainly vary by condition and patient characteristics. The distinct cardiac nature of these ACS patients' clinical encounters may have biased both patient and provider reports, since certain conditions such as chronic pulmonary disease or congestive heart failure were more likely assessed by providers during each patient's hospital admission.

Our original hypothesis that medical records data would reflect a general underreporting of certain conditions that providers considered to be less pertinent to patients' ACS admitting diagnoses appears to be generally supported. Our hypothesis that non-cardiac conditions would be underreported by providers due to patients' admission diagnoses does appear to be supported if we accept patient self-reports as more comprehensive.

These findings specifically demonstrate that for patients who are older or have depressive symptoms, the agreement between data obtained from the two reporting sources may also be significantly affected. Although the practice of including family members could introduce potential response biases, having family members serve as co-informants during research interviews may help older and/or depressed patients recall a large proportion of their earlier diagnosed conditions to improve reporting.

We could conclude similar to earlier studies [10,22] that patients with smaller numbers of self-perceived conditions to recall during interviews may have reported a larger proportion of their total documented comorbidities to increase concordance with medical records. Although the generalizability of these results to non-cardiac or higher comorbidity samples may be limited, our results still suggest that discordance levels between these two sources may be even higher for sicker patient groups with multiple admission diagnoses.

The apparent influence of patients' level of completed education was inconsistent across different conditions. For example, Table 4 indicates that better educated patients are less likely to report a prior AMI or congestive heart failure that is not in the medical record, but are more likely to report cancer that is not documented. This may well be an indication that better educated patients offered more reliable information, if we are willing to accept the assumption that the medical records were more accurate on cardiac-related diseases, but not on less relevant diseases such as cancer. Wording refinements during self-report interviews of some technical medical terms still in the Katz [21] method may facilitate more accurate responses from patients with lower health literacy or education.

Our analyses may have been affected by several limitations. The majority of these post-discharge data came from a sample of hospitalized ACS patients with relatively few reported comorbidities in a specific area of the Midwest. Our use of hospital medical records may have limited our ability to capture the entirely of patients' earlier documented conditions. Wording differences between the self-report source (i.e. before your hospitalization) and medical record source (developed to capture both current and past conditions) items may account for some discordance. Since we lacked self-report comorbidity data from non-baseline interviewed patients, we were unable to compare self-reporting patterns of interviewed and non-interviewed sample subgroups.

## Conclusion

Similar to several earlier studies, [2-4,17] our findings strongly suggest that using patient self-report comorbidity reporting sources may be preferable for conditions more likely to affect patients' routine quality of life and functional status (e.g. *connective tissue disorders*, arthritic conditions, diabetes, stomach ailments). For these conditions, the gold standard measurement issue remains problematic in most studies for the prediction of patient health outcomes. Several research groups have suggested, however, that the parallel use of comorbidity data collection methods from both sources may be necessary to analyze or predict some types of health outcomes [5,9,11,14,18]. Although the data provided by patients' significant others or the use of carefully worded comorbidity checklists may be subject to response biases, such methods may help prompt increased recall of past or longstanding comorbid conditions to augment providers' hospitalization documentation.

Additional testing of the factors influencing relative concordance between patient and provider comorbidity data sources may enable researchers to develop more rigorous comorbidity data reporting methods, particularly for heavily comorbid patients. These results support the conclusion made by earlier researchers that further adjustments in comorbidity data collection methods will be required to obtain the fullest comorbidity data to validly predict patient health outcomes [2,4,11,16].

Due to a variety of patient and setting-level factors, studies testing the influences of comorbidities on subsequent patient outcomes will continue to be complex. Judging from our results, additional testing of reporting methods to generate more accurate comorbidity data will be required for distinct patient subgroups. Although these findings indicate that patient self-report methods may provide a feasible source of ACS patient data for some comorbid conditions, the factors contributing to discordance between reporting sources as we have identified will need to be more fully investigated for future outcomes research.

## Abbreviations

ACS: Acute Coronary Syndrome; AMI : Acute Myocardial Infarction; CCI: Charlson Comorbidity Index.

## Availability and requirements

Clinical Trials URL: 

## Competing interests

The author(s) declare that they have no competing interests.

## Authors' contributions

WC conception and design, drafting and revising manuscript. AS conception and design, analysis and interpretation of data, revising manuscript. AO conception and design, revising manuscript. MS conception and design, revising manuscript. PP acquisition of study data. MH-R conception and design, revising manuscript.

## Pre-publication history

The pre-publication history for this paper can be accessed here:


